# Surface-Codes-Based Quantum Communication Networks

**DOI:** 10.3390/e22091059

**Published:** 2020-09-22

**Authors:** Ivan B. Djordjevic

**Affiliations:** Department of Electrical and Computer Engineering, University of Arizona, Tucson, AZ 85721, USA; ivan@email.arizona.edu; Tel.: +1-520-626-5119

**Keywords:** quantum key distribution (QKD), quantum communications networks (QCNs), quantum communications, entanglement, surface codes

## Abstract

In this paper, we propose the surface codes (SCs)-based multipartite quantum communication networks (QCNs). We describe an approach that enables us to simultaneously entangle multiple nodes in an arbitrary network topology based on the SCs. We also describe how to extend the transmission distance between arbitrary two nodes by using the SCs. The numerical results indicate that transmission distance between nodes can be extended to beyond 1000 km by employing simple syndrome decoding. Finally, we describe how to operate the proposed QCN by employing the software-defined networking (SDN) concept.

## 1. Introduction

Quantum information processing (QIP) opens up new avenues for reliable communications, high-precision sensing, and high-performance computing [1,2,3,4,5,6,7,8,9,10,11,12,13,14,15,16,17,18,19,20]. Entanglement represents a unique resource for QIP, which allows quantum computers to solve classically intractable problems [7], provides certifiable security [2] for data transmissions, and enables sensors to achieve measurement sensitivities beyond the classical limit [8]. The quantum communication is the key cornerstone to fully exploit the properties of entanglement. The modern classical communications tend to use heterogeneous networks capable of simultaneous data transmission between nodes connected via different types of channels, such as free-space optical (FSO) and fiber-optics links. Nodes in existing quantum communication networks (QCNs), however, have been limited to a single optical medium. Moreover, trusted node assumption [4] is required to operate the current QCNs. As a result, one compromised node in a QCN can undermine the security of the entire QCN. Several quantum key distribution (QKD) testbeds have been reported so far, such as the DARPA QKD network [5], Tokyo QKD network [6], and the secure communication based on quantum cryptography (SECOQC) network [7]. Unfortunately, these different QKD networks employ the dark fiber infrastructure.

In this paper, we propose the multipartite heterogenous QCN employing the surface codes, which does not require the trusted node assumption. The research on multipartite entanglement is getting momentum with numerous experimental demonstrations, such as [8]. The surface codes, typically defined on a 2-D lattice, are closely related to the quantum topological codes on the boundary [1], introduced by Bravyi and Kitaev [11,12]. This class of codes is highly popular in quantum computing [13,14,15] because only local qubits are involved in stabilizers. In *Litinski’s framework* [14], the surface code for quantum computing is represented as a game, played on a board partitioned in a certain number of tiles. On each tile we can place a logical qubit, represented as a *patch*. The edges of qubits represent the logical Pauli operators [1]. The logical qubits correspond to the surface code (SC) patches. By placing the SC patches in nodes of a communication network, and connecting the neighbouring patches by *d* wavelength channels, corresponding to the distance of the underlying surface code, we can create the quantum communication network. The SC patches placed in intermediate nodes can be operated as the SC-based quantum repeaters, thus extending significantly the transmission distance. When the patch edges in the tiles of neighbouring network nodes are different, we can perform the product measurements to entangle them. For instance, the product *Z*⊗*Z* between adjacent nodes’ patches can be simultaneously measured to introduce the entanglement between two adjacent quantum nodes. Namely, we start with the state |++〉 = 0.5(|00〉 + |11〉 + |01〉 + |10〉) and perform the measurement on *Z*⊗*Z* operator. If the result of the measurement is +1, the qubits end up in state 2^−1/2^(|00〉 + |11〉); otherwise, they end up in state 2^−1/2^ (|01〉 + |10〉). In either case, the qubits are maximally entangled. This indicates that the proposed SC-based QCN is highly flexible and have numerous applications, including: (i) to teleport quantum states between any two nodes in the network, (ii) to develop the information infrastructure with unprecedented security level, (iii) to enable distributed quantum computing, and (iv) to enable ultra-high precision for quantum sensing applications. To operate such a quantum network, we propose to employ the software-defined networking (SDN) concepts.

The paper is organized as follows. In Section 2, we introduce the surface codes and describe briefly the *Litinski’s* formalism needed in incoming sections. In Section 3, we describe the proposed SC-based QCN concept. In Section 4, we describe our approach to extend the transmission distance between QCN nodes. In Section 5, we provide illustrative numerical results. In Section 6, we describe how to operate the proposed SC-based QCN by utilizing the SDN concepts. Finally, in Section 7, we provide some important concluding remarks.

## 2. Surface Codes for Quantum Networking and Distributed Computing

**Figure 1:**
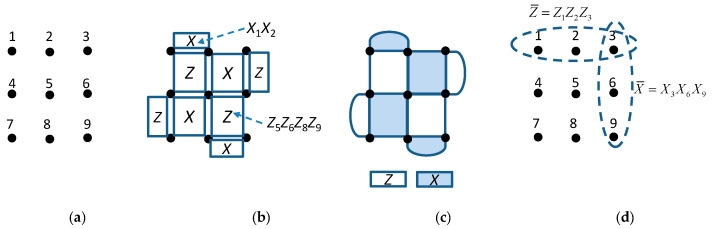
Illustration of a surface code: (**a**) the qubits are located in the lattice positions, (**b**) all-X and all-Z plaquette operators, (**c**) popular representation of surface codes in which stabilizers are clearly indicated, and (**d**) logical operators.

The surface code belongs to the class of topological codes [1] and it is defined on a 2-D lattice, with one illustrative example provided in Figure 1, with qubits being clearly indicated in Figure 1a. The stabilizers of plaquette type can be defined as provided in Figure 1b. Each plaquette stabilizer denoted by X (*Z*) is composed of Pauli *X* (*Z*)-operators on qubits located in the intersection of edges of corresponding plaquette. As an illustration, the plaquette stabilizer denoted by *X* related to qubits 1 and 2 will be *X*_1×2_. The plaquette stabilizer denoted by *Z*, related to qubits 5, 6, 8, and 9, will be *Z*_5_*Z*_6_*Z*_8_*Z*_9_. To simplify the notation, we can use the representation provided in Figure 1c, where the shaded plaquettes correspond to all-*X* containing operators’ stabilizers, while the white plaquettes correspond to all-Z containing operators’ stabilizers. The stabilizers require only local qubits’ interaction, which is not true for other classes of quantum error correction codes.

The weight-2 stabilizers are allocated around perimeter, while weight-4 stabilizers are located in the interior. The logical operators for this code are run over both sides of the lattice, as shown in Figure 1d, and can be represented as $\bar{X}=X_{3}X_{6}X_{9},\;\bar{Z}=Z_{1}Z_{2}Z_{3}$. The codeword length is determined as the product of side lengths, expressed in number of qubits, and, for the surface code from Figure 1, we have that *n* = *L*_x_ × *L*_z_ = 3 × 3 = 9. On the other hand, the number of information qubits is *k* = 1. The minimum distance of this code is determined as the minimum side length, that is *d* = min(*L*_x_,*L*_z_) = 3, indicating that this code can correct a single qubit error.

**Figure 2:**
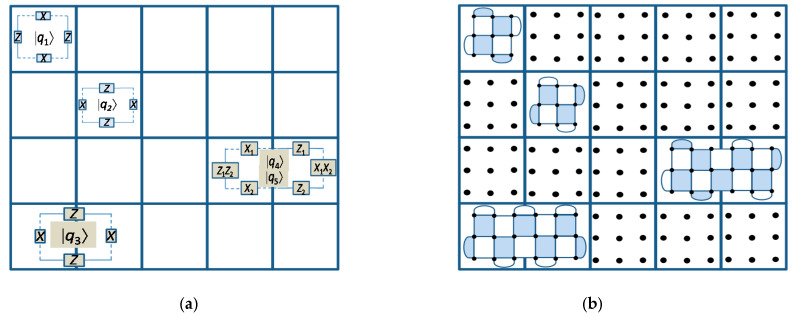
Illustration of one-qubit and two-qubits patches: (**a**) notation and (**b**) actual physical implementation.

**Figure 3:**
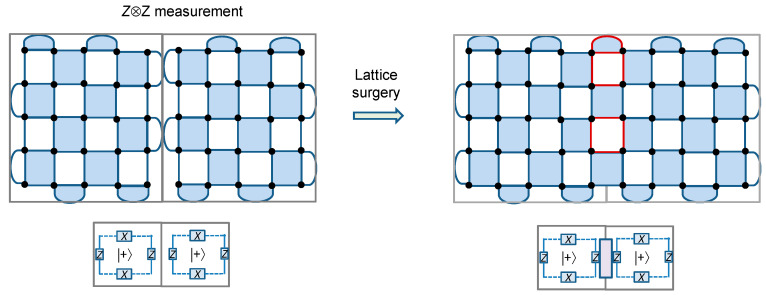
Illustrating the lattice surgery procedure for the measurement on the product Z⊗Z.

Let us now specify the *rules of the game*, that is the operations that can be applied to the patches (qubits), which can be categorized as [14]: (i) *initialization*, (ii) *qubit measurements*, and (iii) *patch deformations*. Compared to computing only limited number of operations are required in quantum networking. With each of these operations, we associate the cost, expressed in terms of time-steps, with each time-step (t.s.) corresponding to ~*d* code cycles (related to the measuring all stabilizers *d* times), with *d* being the distance of underlying surface code per tile. One-qubit patches, shown in Figure 2 as |*q*_1_〉 and |*q*_2_〉, can be initialized to |0〉 or |+〉 states, while two-qubit patches, shown in Figure 2 as |*q*_3_〉, to |00〉 or |++〉 states, with associated cost being 0 t.s. (The logic |+〉-state indicates that all physical qubits are initialized into |+〉-state.) In principle, one-qubit patches can be initialized to the arbitrary states, such as the *magic state* |*m*〉 = |0〉 + exp(jπ/4)|1〉; however, an undetected Pauli error [1] can spoil the initialized state. The *single-patch measurements* can be performed in *X* or Z bases, and after the measurement the corresponding patches get removed from the board, thus freeing up the occupied tiles for future use. The cost associated with single-patch measurements is 0 t.s. For *two-patch measurements*, when the edges in neighboring tiles are different, we can perform the product measurements. As an illustration, the product *Z*⊗*Z* between adjacent patches can be measured as illustrated in Figure 3 (left).

In surface codes, this corresponds to the *lattice surgery* [14,15], in which we change the configuration as shown in Figure 3 (right) by introducing the patches with dark-red edges; after that, we measure the stabilizers for *d* cycles to get the outcome of measurements, and split again. The cost associated with the lattice surgery is 1 t.s. This represents the way to introduce the entanglement between two adjacent patches. Namely, we start with the state |++〉 = 0.5(|00〉 + |11〉 + |01〉 + |10〉) and perform the measurement on *Z*⊗*Z* operator. If the result of the measurement is +1, the qubits end up in state 2^−1/2^(|00〉 + |11〉); otherwise, they end up in state 2^−1/2^ (|01〉 + |10〉). In either case, the qubits are maximally entangled. We can apply the similar procedure to the *X*⊗*Z* product operator. Of course, it is also possible to measure the product operator involving the *Y* operator, which is really not needed in our proposed QCNs. What is even more interesting is that it is possible to measure the product for more than two encoded Pauli operators through *multi-patch measurements* [14].

## 3. Proposed Surface-Codes-Based Quantum Communications Networks

**Figure 4:**
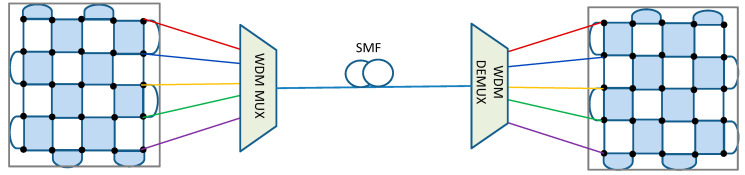
Simplified description of connecting two logical qubits from two neighboring nodes by *d* wavelengths.

To enable the next generation of quantum communication networking, we propose to employ the surface codes so that the logical qubits are located at different nodes in the network. The logical qubits are represented by the patches introduced in the previous section. For simplicity, we assume that the surface code is defined on a *d* × *d* grid. The neighboring nodes are connected by employing *d* wavelengths, as illustrated in Figure 4. Some of the optical links could be FSO links.

**Figure 5:**
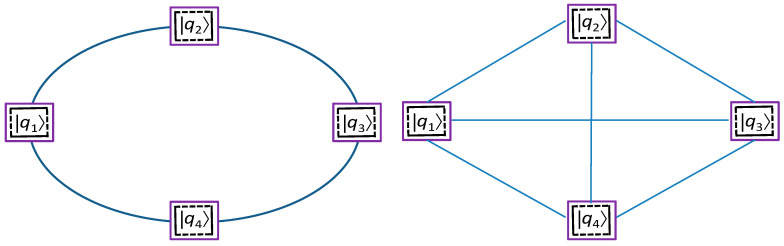
Illustrative four-node ring (**left**) and four-node mesh (**right**) quantum communication networks.

By performing the *Z*⊗*Z* measurement, as described in the previous section, the logical qubits create the Einstein–Podolsky–Rosen (EPR) pair. The results of the measurements have been passed to the SDN controller, which will know the exact EPR pair being created. To create the desired QCN, the corresponding product measurements need to be simultaneously performed. As an illustration, let us consider the ring network composed of four nodes, as shown in Figure 5 (left), with each node being equipped with the surface code patch representing the corresponding logical qubits. In principle, one surface patch can be split between multiple nodes, but, to facilitate explanations, we assume that each node contains a single SC patch. By performing the simultaneous product *Z*⊗*Z* measurements between logical qubits *q*_1_ and *q*_2_, *q*_2_ and *q*_3_, *q*_3_ and *q*_4_, *q*_4_ and *q*_1_, we can entangle the nodes 1–4 and thus create the ring QCN. On the other hand, for the four-node mesh network shown in Figure 5 (right), by performing the simultaneous product *Z*⊗*Z* measurements between logical qubits *q*_1_ and *q*_2_, *q*_2_ and *q*_3_, *q*_3_ and *q*_4_, *q*_4_ and *q*_1_ as well as the simultaneous *X*⊗*X* measurements between *q*_1_ and *q*_3_, *q*_2_ and *q*_4_, we can entangle the four qubits into the mesh configuration. By providing the results of the measurements to the SDN control plane, the exact maximum entangled state between nodes in the QCN will be known. Clearly, this approach allows us to entangle the logical qubits in an arbitrary network. The trapped ions-based technology represents a perfect candidate for practical implementation of the proposed QCN. By equipping every node in the proposed QCN by multiple qubit patches, in principle, we can simultaneously perform quantum networking and quantum distributed computing. Instead of wavelength-division multiplexing (WDM), the multicore fiber can also be used to connect the logical qubits [16]. The proposed QCN does not require the trusted node assumption, but it is assumed that Eve does not have access to SDN controller.

## 4. Extending the Distance between the Nodes in the Proposed SC-Based QCN

**Figure 6:**
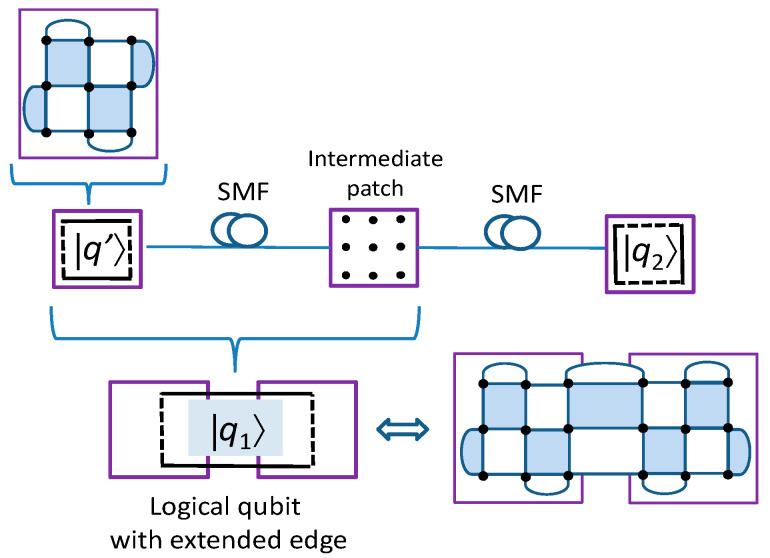
Extending the distance between two nodes in a quantum communication network (QCN) by creating the logical qubit spanning two spatially separated surface code (SC) patches.

To extend the transmission distance between neighboring nodes in QCN, we propose to use the *quantum error correction (QEC)-based repeaters*. So far, QEC-based repeaters are based on two-dimensional QE-based repeaters, such as the dual-containing Calderbank–Shor–Steane (CSS)-codes-based repeaters [17] and surface-codes-based repeaters [18]. Unfortunately, dual-containing CSS codes are essentially girth-4 quantum low-density parity-check (LDPC) codes with poor error correction performance [19]. On the other hand, the surface codes proposed in [18] introduce large latency and are not compatible with the QCN proposed in the previous section. Here, we propose a different approach to interpret an intermediate node as an SC patch and apply the patch deformation approach due to Litinski and thus extend the logical qubit to two spatially separated patches, which is illustrated in Figure 6. In this example, three wavelengths are needed to interact remote patches. Once the logical qubit is extended to the intermediate node, we further perform product *X*⊗*Z* measurements to entangle the logical qubits *q*_1_ and *q*_2_. This approach is applicable to several intermediate nodes, thus offering the potential to significantly extend the distance between any two desired nodes in the QCN.

## 5. Illustrative Numerical Results

**Figure 7:**
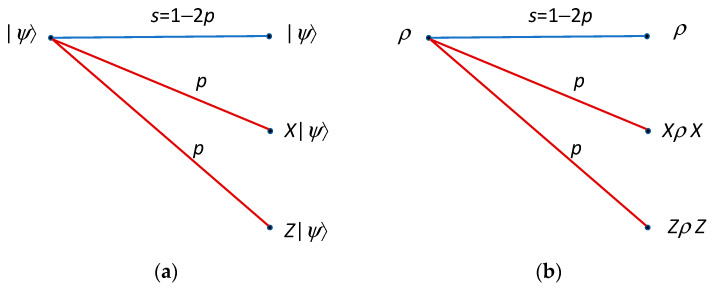
Quantum channel model under study: (**a**) Pauli operator description and (**b**) density operator description.

Although the channel loss dominates the performance of quantum repeaters, there will be quantum errors associated with each stage, which can be represented by using the quantum channel model provided in Figure 7, where *X* and *Z* quantum errors occur with the same probability *p*. The corresponding Kraus representation [1] is given by:

(1) $$\rho_{f}=\xi \left(\rho \right)=\left(1-2p\right)\rho +pX\;\rho X+pZ\;\rho Z.$$

Let us consider the BB84 protocol by employing the approach introduced in previous section. The corresponding secret-key rate after *N* sections will be:

(2) $$SKR={\left\{\left[1-P\left(E\right)\right]T\right\}}^{N}\max\left(1-h_{2}\left(q_{N}^{\left(Z\right)}\right)-f_{e}h_{2}\left(q_{N}^{\left(X\right)}\right),0\right),$$

where *f_e_* denotes the error correction inefficiency (*f_e_ ≥* 1), $q_{N}^{\left(X\right)}\;\left[q_{N}^{\left(Z\right)}\right]$ denotes the quantum bit-error rate (QBER) in the *X*-basis (*Z*-basis) after *N* stages, *T* represents the single link transmissivity, and *h*_2_(*x*) is the binary entropy function $h_{2}\left(x\right)=-x\log_{2}\left(x\right)-\left(1-x\right)\log_{2}\left(1-x\right)$. The term $h_{2}\left(q_{N}^{\left(Z\right)}\right)$ represents the amount of information Eve was able to learn during the raw key transmission, which can be removed from the final key during the privacy amplification phase. The term $f_{e}h_{2}\left(q_{N}^{\left(X\right)}\right)$ represents the amount of information revealed to Eve during the information reconciliation stage. The dark counts, device imperfections, and errors introduced by Eve are all contributed to the Eve and included in transition probability *p*. The QBER after *N* stages can be estimated by:

(3) $$q_{N}^{}=\frac{1-s^{N}}{2},\;s=1-2p.$$

The probability of the syndrome decoding error is bounded by [1]:

(4) $$P\left(E\right)\leq \sum_{j=\left\lfloor \left(d-1\right)/2\right\rfloor +1}^{d^{2}}\left(\begin{array}{c} d^{2}\\ j \end{array}\right){\left(1-s\right)}^{j}s^{d^{2}-j},\;s=1-2p,$$

**Figure 8:**
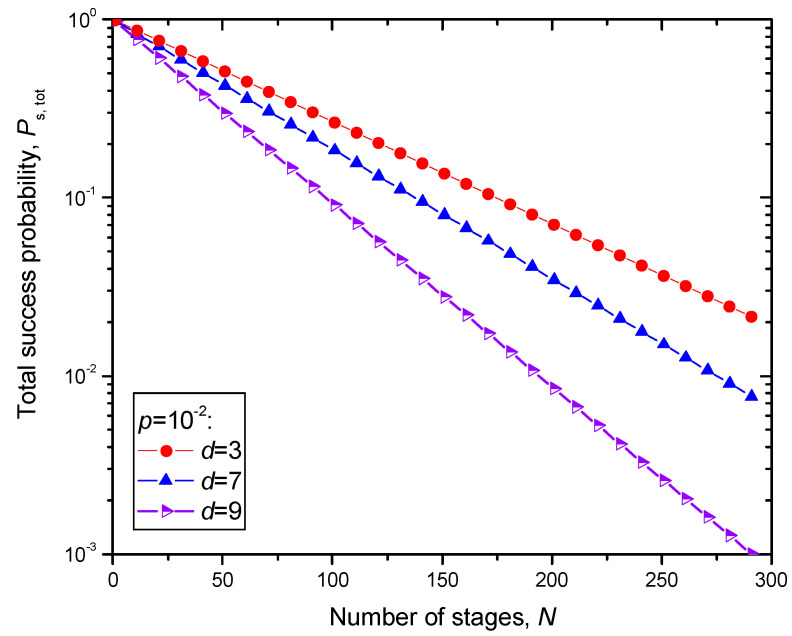
Total success probability defined as (1 − *P*(*E*))*^N^*, where *N* is the number of stages, when the *d* × *d* surface code is used, and syndrome decoding is applied.

So, [1 − *P*(*E*)]*T* represents the success probability for the single stage. The total success probability can be estimated by {[1 − *P*(*E*)]*T*}*^N^* and is illustrated in Figure 8 by setting the *X* (*Z*) qubit error probability to *p* = 10^−2^ and transmissivity to *T* = 1, for different *d* × *d* surface codes.

**Figure 9:**
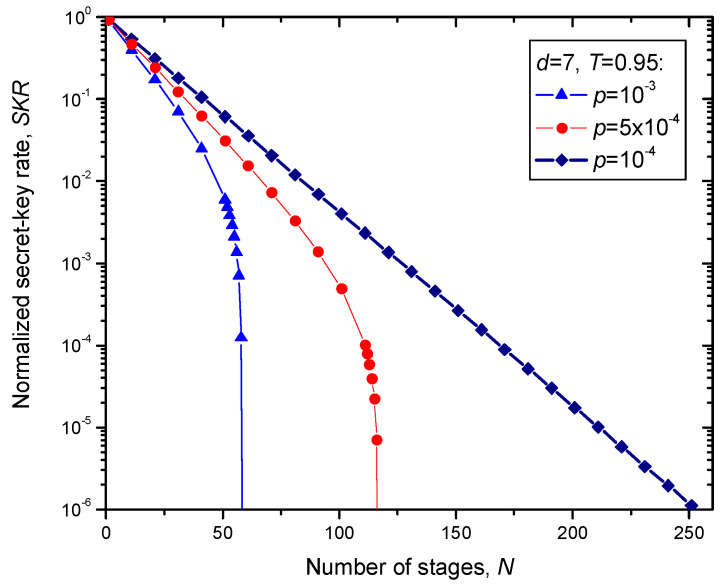
Normalized secret-key rate (SKR) vs. the number of stages *N* assuming that the 7 × 7 surface code is used, and single link channel transmittance is *T* = 0.95.

**Figure 10:**
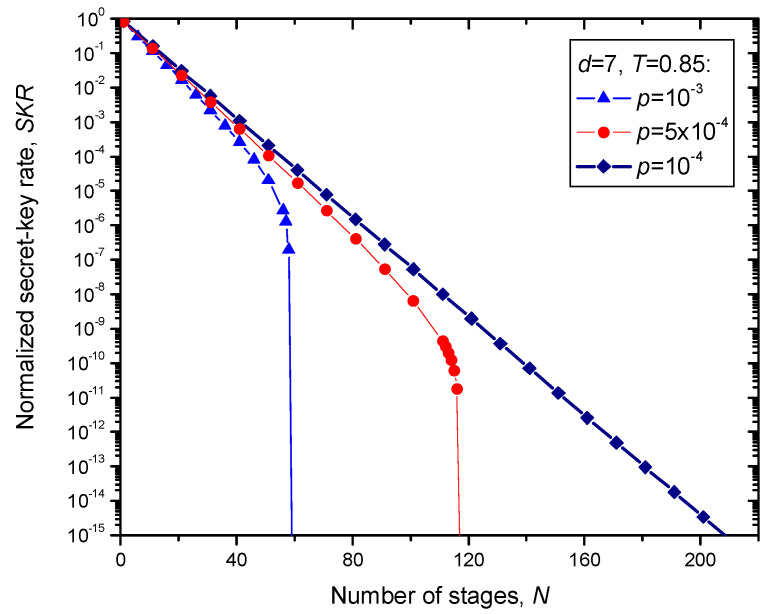
Normalized SKR vs. number of stages *N* assuming that 7 × 7 surface code is used, and single link channel transmittance is *T* = 0.85.

The numerical results for secret-key rate (SKR) for different transmissivities *T* (assuming that *f_e_* = 1) vs. the number of stages *N* are summarized in Figure 9 and Figure 10. The channel transmittance in Figure 9 is set to *T* = 0.95, while in Figure 10 it is set to *T* = 0.85. The qubit error transition probability *p* is used as a parameter. In both figures, the 7 × 7 surface code is used. Given that the effective transmission distance of the fiber is given by [20]:

(5) $$L_{\mathrm{eff}}=\frac{1-e^{-\alpha L}}{\alpha }\approx 1/\alpha ,$$

For ultra-low loss fiber introduced in [21] with attenuation coefficient α = 0.1419 dB/km, we obtain that *L*_eff_ = 30.606 km. The total transmission length can be now estimated by:

(6) $$L_{\mathrm{tot}}=NL_{\mathrm{eff}}\left|\ln T\right|.$$

For *T* = 0.95, by setting the qubit error probability to *p* = 10^−4^, we can see from Figure 9 that the achievable total transmission distance for normalized SKR of 10^−6^ is *L*_tot_ = 252 × 30.606 × |ln0.95| = 395.61 km. On the other hand, for *T* = 0.85, by setting the qubit error probability to *p* = 10^−4^, we can see from Figure 10 that the achievable total transmission distance for normalized SKR of 10^−15^ (typical for discrete variable QKD schemes [2]) is *L*_tot_ = 208 × 30.606 × |ln0.85| = 1034.61 km, and this results is comparable to the recently proposed hybrid QKD-postquantum cryptography scheme [22,23]. By employing higher complexity quantum sum-product algorithm [1] in each stage, instead of simple syndrome decoding, the total transmission distance well beyond 1000 km can be achieved. Typical QKD transmission distances are significantly shorter, even when the most advanced twin-field QKD schemes are used [24].

## 6. Operating the Proposed QCN by SDN Control

The SDN has been introduced to separate the control plane and data plane, manage network services through abstraction of higher-level functionality, and implement new applications and algorithms efficiently [25,26]. It has already been studied to enable the coexistence of classical and quantum communication channels [27]. To enhance the security of the software-defined optical networks, authors in [28] proposed a four-layer architecture composed of: application, control, QKD, and data layers. The SDN-based QCN architecture compatible with the proposed QCN should contain three layers only—namely, application layer, control layer, and QCN layer. Users will send their requests from the application layer with the help of northbound interface to the SDN controller. The SDN controller will allocate the QCN resources with the help of its global map through the southbound interface. The QCN layer can be composed of DWDM links and QCN nodes. Each QCN node should contain quantum transceivers, integrated on the same chip, together with a *d* × *d* array of physical qubits. Any two nodes in QCN can communicate through either a dedicated SMF link or by *d* wavelength channels. To enable so, we could employ our recently proposed bidirectional optical space switch [29], to reconfigure the QCN. Other alternative optical switches can be used as well. In addition to conventional modules, the application layer should also have modules to provide security management services. On the other hand, the control layer, in addition to controlling the QCN layer, should provide allocation of resources as well as provide services for multiple applications. To deal with time-varying channel conditions over heterogeneous links, we can adapt the channel configuration based on both application requirements and link conditions.

## 7. Concluding Remarks

To enable the next generation of quantum communication networks, we have proposed to employ the surface-codes-based patches as quantum nodes. We have described how to simultaneously entangle multiple quantum nodes in any quantum network topology by employing the SCs. We have also described how to extend the transmission distance between any two quantum nodes to beyond 1000 km. Finally, we have described how to operate the proposed QCN by employing the SDN concept. The trapped ion technology is an excellent candidate to be used as an enabling technology to implement SC-based QCNs. One important issue will be to implement a portable, rack-mounted ion-trap-based quantum interface, and some progress has already been made by researchers from Duke University in collaboration with ColdQuanta, Inc [30]. To improve the efficiency of the proposed QCNs, the high-dimensional SCs should be employed. By employing high-dimensional-based quantum error correction, we can achieve error correction capability comparable to 2D but with significantly shorter codeword lengths as discussed in [31]. An alternative approach to the proposed QCN will be a recently introduced cluster-state-based QCN [32].
